# Acetylated Globotetraose (Ac-Gb4) Suppresses Triple-Negative Breast Cancer Through FAK/AKT Signaling Pathway

**DOI:** 10.3390/ijms252413353

**Published:** 2024-12-12

**Authors:** Yung-Kuo Lee, Misbah Sehar, Lavanya Botcha, Po-Kai Chuang

**Affiliations:** 1 Medical Laboratory, Medical Education and Research Center, Kaohsiung Armed Forces General Hospital, Kaohsiung 80284, Taiwan; 2 Institute of Medical Science and Technology, National Sun Yat-Sen University, Kaohsiung 80424, Taiwan; 3 Institute of Biomedical Sciences, National Sun Yat-Sen University, Kaohsiung 80284, Taiwan

**Keywords:** acetylated globotetraose, triple-negative breast cancer, SSEA3, SSEA4, FAK/AKT signaling

## Abstract

Triple-negative breast cancer (TNBC) remains a significant therapeutic challenge due to its unresponsiveness to hormone and HER2-targeted treatments. This study investigated the potential of acetylated globotetraose (Ac-Gb4) as a novel therapeutic approach targeting glycolipid-mediated signaling in breast cancer cells. We synthesized acetylated globotetraose (Gb4) to enhance its membrane permeability while preserving its biological recognition properties. Flow cytometry analysis revealed that Ac-Gb4 treatment significantly decreased SSEA3 and SSEA4 expression in MDA-MB-231 breast cancer cells, which are Globo-H-negative cells. Notably, Ac-Gb4 demonstrated selective cytotoxicity against cancer cells by significantly reducing proliferation and inducing apoptosis in MDA-MB-231 cells while sparing hTERT-HME1 normal breast epithelial cells. Mechanistic studies through Western blot analysis revealed that Ac-Gb4 simultaneously modulated multiple signaling pathways, including FAK cleavage, reduced AKT expression, and increased caspase-3 activation, particularly at the 4 mM concentration. These molecular changes correlated with decreased cancer cell invasion capability in a dose-dependent manner. Our findings demonstrated that Ac-Gb4 effectively targeted breast cancer cells through the modulation of critical signaling pathways involved in cell survival and invasion while maintaining a minimal impact on normal cells. This anti-cancer activity suggests that Ac-Gb4 represents a promising therapeutic candidate for breast cancer treatment, particularly for aggressive subtypes such as TNBC.

## 1. Introduction

Breast cancer continues to pose a major global health burden, affecting millions and representing the most common form of cancer among women [1]. According to the World Health Organization, breast cancer accounts for 24.5% of all cancers in women and is a leading cause of cancer-related mortality worldwide, with over 680,000 deaths reported in 2020 alone [2]. Despite significant advancements in early detection and treatment options, such as surgery, chemotherapy, radiotherapy, and hormone and targeted therapies, breast cancer remains a major challenge due to high recurrence rates and the prevalence of aggressive, treatment-resistant subtypes, particularly triple-negative breast cancer (TNBC) [3,4]. TNBC is notably challenging to treat due to its lack of estrogen and progesterone receptors and the absence of HER2 amplification, which renders it unresponsive to hormone therapies and HER2-targeted treatments [5]. As such, there is an urgent need for new therapeutic agents that selectively target cancer cells while minimizing damage to normal tissue, especially for aggressive subtypes such as TNBC.

Among various molecular targets being explored in breast cancer research, glycolipids carbohydrate-containing lipid molecules embedded within the cell membrane have garnered attention for their role in cellular adhesion, signaling, and immune response [6,7]. Glycolipid regulation in breast cancer significantly influences multiple cellular processes critical for cancer development and progression [8]. The altered expression of glycolipids on breast cancer cell surfaces modifies cellular recognition, adhesion properties, and signal transduction pathways, thereby affecting cancer cell behavior and treatment responses [9,10]. Expression patterns of specific glycolipids, including globo-series (SSEA3, SSEA4, and Globo-H) and gangliosides, undergo significant alterations during breast cancer development, leading to profound effects on cellular function and behavior [11,12,13]. The modification of glycolipid profiles substantially impacts cancer cell interactions with the extracellular matrix and other cells, directly influencing metastatic potential and tissue invasion capabilities [14]. Furthermore, alterations in membrane glycolipid composition significantly affect receptor-mediated signaling pathways, particularly those involving growth factor receptors and cell survival mechanisms, thereby modulating cancer cell proliferation, metastasis, and evasion of apoptosis [15,16].

Specifically, the globotetraose (Gb4) glycolipid, a member of the globo-series glycolipids, has been implicated in oncogenic processes and is frequently overexpressed in several malignancies, including breast cancer [17]. The conversion of globotetraose (Gb4) to SSEA3 (Gb5) involves a specific enzymatic pathway in the globo-series glycosphingolipid biosynthesis [18,19]. Beta-1,3-galactosyltransferase 5 (B3GALT5) catalyzes this crucial step by transferring a galactose residue to Gb4 through a β1,3-linkage. B3GALT5 transfers galactose from UDP-galactose to the terminal N-acetylgalactosamine (GalNAc) residue of Gb4 (GalNAcβ1-3Galα1-4Galβ1-4Glcβ1-1Cer) to form SSEA3 (Galβ1-3GalNAcβ1-3Galα1-4Galβ1-4Glcβ1-1Cer) [20]. This enzymatic addition extends the carbohydrate chain and creates the specific structure recognized as the SSEA3 epitope. The formation of SSEA3, SSEA4, and Globo-H through this pathway represents a critical step in globo-series glycosphingolipid metabolism, as SSEA3 serves as a precursor for more complex globo-series structures and plays important roles in cellular recognition and signaling processes [21]. However, we hypothesized that additional supplementation of Gb4 to cells could lead to enzymatic exhaustion as a potential approach, potentially reducing downstream SSEA3 synthesis and thereby inhibiting cancer progression.

Therefore, the chemical modification through acetylation represents a strategic approach to enhance cellular membrane permeability [22]. Acetylation alters the physicochemical properties of Gb4 by adding acetyl groups to available hydroxyl positions in the molecule structure, thereby increasing molecular lipophilicity [23]. Glycosphingolipids, particularly Gb4 and SSEA3, play crucial roles in cellular signaling pathways that regulate cell proliferation and invasion. These glycolipids interact with membrane-associated signaling complexes, notably affecting the Focal Adhesion Kinase (FAK) and AKT pathways, which are critical mediators of cellular survival, proliferation, and invasion processes [9]. FAK signaling represents a key pathway in cell adhesion and invasion mechanisms, where FAK undergoes autophosphorylation upon activation, initiating downstream signaling cascades that regulate cell motility and invasion [24]. Alterations in membrane glycosphingolipid composition, particularly changes in Gb4 and SSEA3 levels, can modulate FAK activation patterns through interactions between membrane glycosphingolipids and integrin complexes, thereby affecting cellular adhesion and invasion capabilities [18]. Additionally, the AKT pathway, central to cell survival and proliferation, demonstrates sensitivity to membrane glycosphingolipid composition, requiring proper membrane organization and lipid raft integrity where glycosphingolipids serve as important structural components [25]. Changes in Gb4 and SSEA3 levels can affect the formation and stability of these membrane microdomains, consequently influencing AKT activation and downstream survival signaling [26,27].

In breast cancer cells, elevated levels of specific glycosphingolipids correlate with enhanced FAK and AKT activation, promoting increased cell survival and invasive potential [21]. Modulation of glycosphingolipid metabolism, particularly through targeting Gb4 and SSEA3 synthesis or turnover, represents a potential strategy for regulating these signaling pathways, providing insights into potential therapeutic approaches targeting cancer cell proliferation and invasion [28]. The development of glycolipid-modifying agents such as Ac-Gb4 represents a promising therapeutic strategy, as it interferes with cell proliferation and metastasis-related pathways through targeting globo-series glycolipids in cancer. We demonstrated that Ac-Gb4 effectively modulates cell membrane properties and disrupts cancer-specific glycolipid expression patterns, thereby affecting cell viability and proliferation, which provides essential insights for therapeutic development in cancer treatment.

## 2. Results

### 2.1. The Potential and Importance of Ac-Gb4 to Reduce B3GalT5 Enzymatic Activity

**Figure 1:**
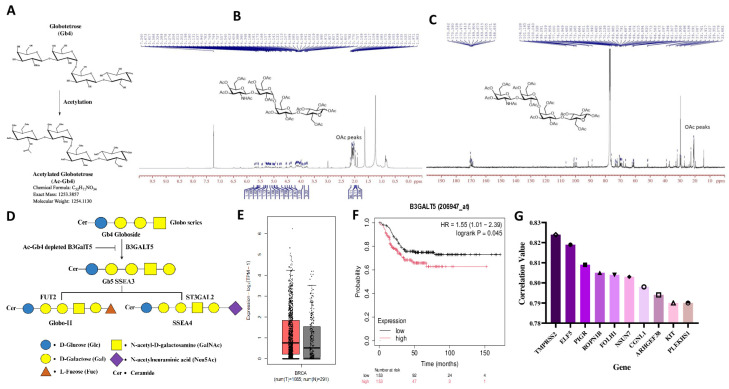
Characterization of Globotetraose (Gb4) acetylation and its targeting B3GalT5 associated gene expression profiling. Schematic representation of globotetraose modification and B3GalT5-associated gene expression analysis. (**A**) Molecular structure of unmodified globotetraose (Gb4) and the structure of acetylated globotetraose (Ac-Gb4) showing acetylation modification. (**B**) ^1^H NMR spectrum of acetylated globotetraose (Ac-Gb4). (**C**) ^13^C NMR spectrum of acetylated globotetraose (Ac-Gb4). (**D**) Synthesis pathway and correlated enzymes from Gb4 to SSEA3 and SSEA4 or Globo-H. We hypothesized that the Ac-Gb4 adds to B3GalT5 enzyme depletion. (**E**) Tumor with high B3GalT5 expression (red: tumor tissue, grey: normal tissue: (GEPIA2 database http://gepia2.cancer-pku.cn/, accessed on 13 November 2024)). Baseline expression levels of B3GalT5 gene expression in patients with breast cancer. (**F**) Disease-free survival indicated that high expression of B3GalT5 decreased the survival rate in breast cancer patients (*p* < 0.045). (**G**) Top 10 differentially expressed genes associated with B3GalT5 gene (total expression value = 8.036), including membrane protease TMPRSS2 (10.25%), transcription factor ELF5 (10.19%), immunoglobulin receptor PIGR (10.07%), motility-associated ROPN1B (10.02%), metabolic enzyme FOLH1 (10.00%), methyltransferase NSUN7 (9.99%), cell junction protein CGNL1 (9.93%), signaling regulator ARHGEF38 (9.88%), tyrosine kinase receptor KIT (9.83%), and signaling protein PLEKHS1 (9.83%). The results reveal the potential possibility of using Ac-Gb4 to suppress the regulatory network between B3GalT5 enzymatic activity and its cellular signaling pathways.

To target glycosphingolipid metabolism in TNBC, we investigated the effect of chemical modification of globotetraose (Gb4) through acetylation. Figure 1A illustrates the structural modification of Ac-Gb4, where acetyl groups are added to hydroxyl positions to increase lipophilicity in the molecule. This acetylation process alters the physicochemical properties of Gb4, leading to improved membrane permeability and the modulation of glycosphingolipid profiles. Therefore, the resultant modified structure exhibits enhanced membrane interaction characteristics while preserving the fundamental glycosphingolipid architecture necessary for biological activity. The structural confirmation of Ac-Gb4 was carried out by nuclear magnetic resonance ^1^H NMR and ^13^C NMR spectroscopy. The ^1^H NMR spectrum (Figure 1B) showed signals corresponding to the sugar protons of the Gb4 backbone. The anomeric protons were observed as 5.2–4.5 ppm, confirming the glycosidic linkages. The acetyl groups were observed at 2.0–2.2 ppm, representing the methyl protons of the acetyl functional groups. The spectrum displayed multiple peaks in the 3.0–4.0 ppm range, corresponding to the protons of the sugar rings. Integration of these signals confirmed the expected stoichiometry of the Ac-Gb4 molecule. No extraneous peaks were detected, which indicates the high purity of the synthesized compound. The ^13^C NMR spectrum of Ac-Gb4 reveals characteristic peaks corresponding to the various carbon environments within the molecule (Figure 1C). Peaks around 170 ppm indicate the carbonyl carbon of the acetyl group, while 22 ppm represents the methyl of the acetyl group. Additional signals around 60–80 ppm correspond to carbon in saturated environments, possibly alkyl chains or carbon adjacent to heteroatoms such as oxygen. The spectrum displays peaks around 30–50 ppm, representing carbon attached to alkyl groups or adjacent to electronegative atoms. The sugar carbons including the globotetraose backbone are observed in the region between 20 and 40 ppm, which confirms the acetylation of the Gb4 molecule. These results demonstrate the successful acetylation of Gb4 and provide a detailed structural characterization of Ac-Gb4. In the globo-series glycosphingolipid biosynthesis pathway (Figure 1D), the B3GALT5 enzyme synthesizes the Gb4 globoside into SSEA3, which further serves as a precursor to form SSEA4 and Globo-H structures. In our study, the bioinformatics analysis of the B3GALT5 gene in breast cancer show that the gene expression is upregulated in the tumor sample highlighted in red when compared to the normal samples, highlighted in gray, that were used for analysis, indicating that the expression of B3GALT5 could be a useful marker in the identification of breast cancer (Figure 1E). To understand the effect of upregulated expression of B3GALT5 on the mortality rate, we then conducted a survival analysis using Kaplan–Meier Plotter (Figure 1F). Triple-negative breast cancer patients with high B3GALT5 expression showed a lower survival rate, indicating that the gene expression has a significant effect on the survival rate. To investigate gene correlations with B3GALT5 in tumor tissue, we performed a correlation analysis and identified the top ten genes using Correlation AnalyzeR, as displayed in Table S4, and represented them through the bar plot in Figure 1G. We then analyzed individual correlations through GEPIA2; we observed the top ten genes with high correlation (Table S4) and ten genes with the least correlation (Table S5) with the B3GALT5 gene and observed that B3GALT5 shows moderate correlation with multiple genes, even while the gene expression is statistically significant such as in the cases of ARHGEF38, ELF5, and FOLH1 (Figure S6). Through this, we can understand that B3GALT5 exhibits minor correlations with approximately three out of the top ten genes that showed correlation.

### 2.2. In Silico Evaluation of Ac-Gb4 and B3GalT5 Interaction

**Figure 2:**
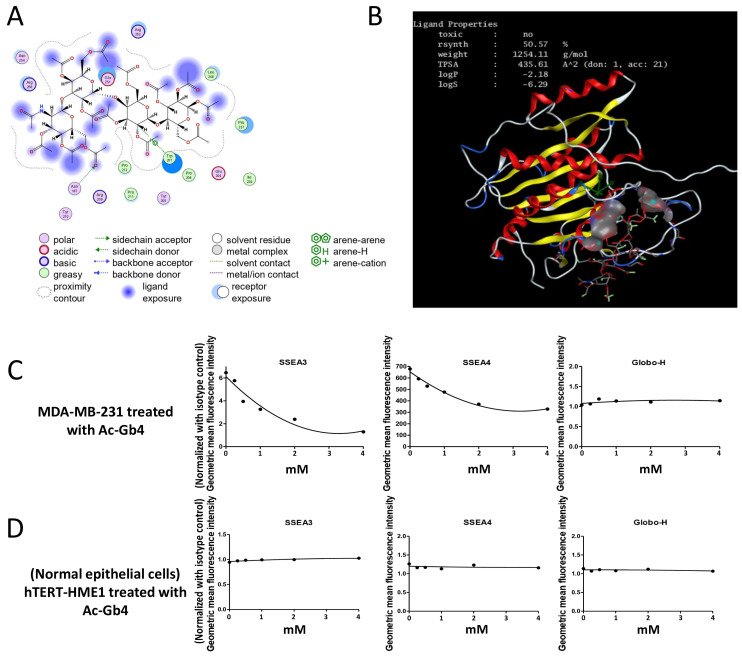
Analysis of Ac-Gb4 Molecular Docking and Its Effects on Glycosphingolipid Expression in Breast Cancer and Normal Cells. Molecular docking analysis of Ac-Gb4 and its differential effects on glycosphingolipid expression. (**A**) Ligand interaction diagram showing the binding interactions between Ac-Gb4 and B3GALT5, including hydrogen bonds, hydrophobic interactions, and solvent exposure. (**B**) Three-dimensional molecular docking model of Ac-Gb4 with B3GALT5 showing ligand properties including TPSA, logP, and logS values. The protein structure is represented in ribbon format with alpha helices (red), beta sheets (yellow), and loops (white). (**C**) Dose-dependent effects of Ac-Gb4 treatment (0–4 mM) on SSEA3, SSEA4, and Globo-H expression in MDA-MB231 breast cancer cells, showing significant reduction in SSEA3 and SSEA4 levels. (**D**) Expression levels of SSEA3, SSEA4, and Globo-H in hTERT-HME1 normal epithelial cells treated with Ac-Gb4 (0–4 mM). The geometric mean fluorescence intensity was normalized to isotype control.

The drug-likeness properties of Ac-Gb4 reveal several favorable characteristics that enhance its potential as a therapeutic candidate for triple-negative breast cancer (TNBC) (Figure S7). Ac-Gb4 demonstrates favorable drug-like properties including good intestinal absorption and CaCo2 permeability with minimal risk of metabolic interaction and toxicity (Table S3). It is non-mutagenic and non-carcinogenic supporting its potential as a safe and effective therapeutic candidate for TNBC. In this study, molecular docking was carried out to explore the interaction between Ac-Gb4 and B3GALT5, which play a role in glycolipid synthesis associated with the cancer signaling pathway. Molecular docking of Ac-Gb4 with B3GALT5 was performed using Molecular Operating Environment (MOE version 2015.10, https://www.chemcomp.com, accessed on 10 August 2024) software (Figure 2A,B and Figure S8). This analysis investigated interaction sites and binding affinity, providing insights into how Ac-Gb4 targets glycolipid synthetic enzyme-mediated pathways. The absorption, distribution, metabolism, excretion, and toxicity (ADMET) profile of Ac-Gb4 was evaluated to assess its drug-like properties. The analysis revealed favorable characteristics, including desirable absorption and metabolism profiles, supporting its potential as a therapeutic agent (Table S6). The binding affinity of Ac-Gb4 was ∆G = −9.3 (Kcal/mol) (Table S7). A careful examination of the ligand–receptor complex revealed diverse interactions between polar residues and arene hydrogen bonds which could contribute to altering the enzyme function and modulate downstream signaling pathways such as FAK/AKT in TNBC.

### 2.3. Globo-Series Glycolipid Expression on Breast Cell Surface

To evaluate the impact of Ac-Gb4 treatment on globo-series glycolipid expression patterns at the breast cell surface, we conducted comprehensive flow cytometric analyses of both MDA-MB-231 breast cancer cells and hTERT-HME1 normal breast epithelial cells. Systematic examination using increasing concentrations of Ac-Gb4 (ranging from 0.25 mM to 4 mM) revealed distinct alterations in cell surface glycolipid profiles. Flow cytometric analysis demonstrated that MDA-MB-231 breast cancer cells exhibited pronounced concentration-dependent decreases in SSEA3 and SSEA4 globo-series glycolipid expression patterns, while Globo-H glycolipid expression remained unchanged (Figure 2C,D). The response pattern in MDA-MB-231 cells showed particularly notable shifts at higher Ac-Gb4 concentrations, suggesting substantial remodeling of the cell surface glycolipids. The flow cytometric data revealed distinct population distributions across the concentration gradient, with marked changes becoming evident at concentrations above 1 mM. These alterations in surface glycolipid expression patterns may reflect fundamental changes in membrane organization and cellular response to Ac-Gb4 treatment. In contrast, hTERT-HME1 normal breast epithelial cells maintained consistent expression patterns of globo-series glycolipids, including SSEA3, SSEA4, and Globo-H, throughout the treatment range. This differential response between malignant and normal cells suggests distinct mechanisms in the incorporation and processing of Ac-Gb4, reflecting fundamental differences in the membrane organization and glycolipid composition between cancer and normal breast epithelial cells.

### 2.4. The Effects of Acetylation on Globotetraose (Ac-Gb4) on Cell Viability

**Figure 3:**
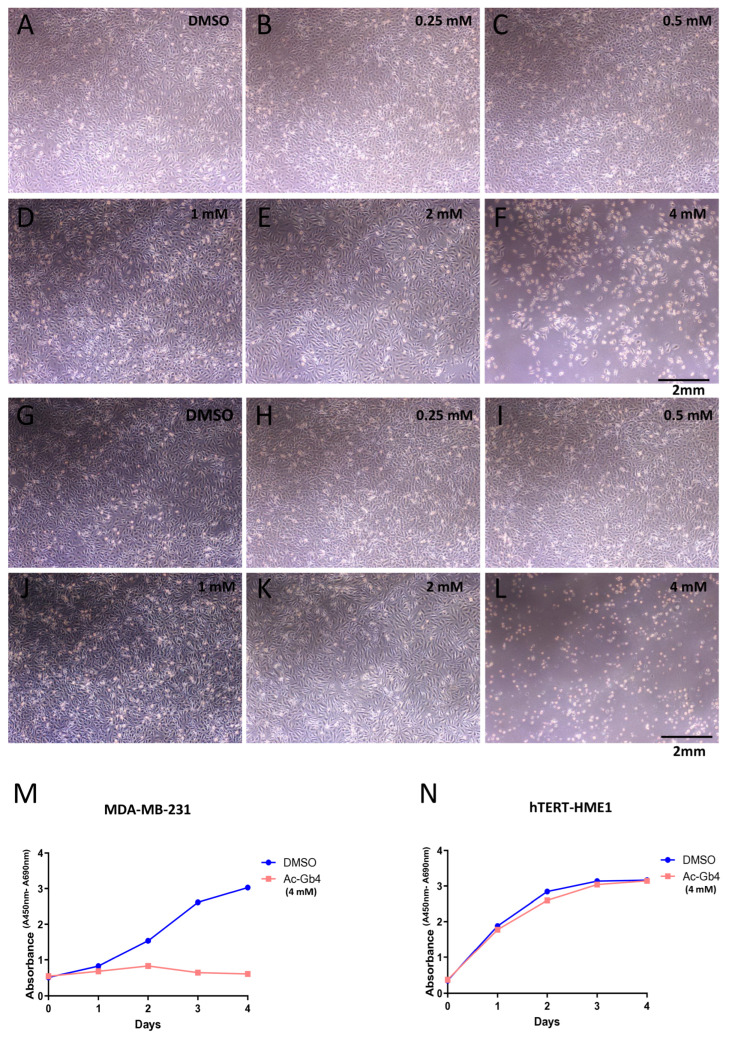
The Effects of Ac-Gb4 on Cell Viability and Proliferation in Breast Cancer and Normal Cells. Analysis of Ac-Gb4 effects on cell proliferation in breast cancer and normal epithelial cells. (**A**–**L**) Representative bright-field microscopy images demonstrating morphological and quantitative alterations in MDA-MB-231 cells after 4 days of exposure to ascending concentrations of Ac-Gb4 (0–4 mM). DMSO was used as vehicle control. Scale bar = 2 mm. (**M**) Cell proliferation assay showing the growth curve of MDA-MB-231 breast cancer cells treated with Ac-Gb4 (4 mM) or DMSO control over 4 days. (**N**) Cell proliferation assay of hTERT-HME1 normal breast epithelial cells treated with Ac-Gb4 (4 mM) or DMSO control over 4 days, demonstrating selective cytotoxicity of Ac-Gb4 against cancer cells while sparing normal cells. Cell proliferation was measured by absorbance at 450nm and 690 nm, *n* = 3.

To investigate the differential effects of Ac-Gb4 on cell viability, we conducted comprehensive proliferation analyses comparing MDA-MB-231 breast cancer cells and hTERT-HME1 normal breast epithelial cells. Treatment of MDA-MB-231 cells with 4 mM Ac-Gb4 resulted in a marked reduction in proliferation compared to that of the DMSO vehicle controls throughout the four-day observation period (Figure 3A,B). Analysis of growth curves demonstrated that Ac-Gb4 induced marked proliferation suppression starting from day 2, with the inhibitory effect maintained through the entire observation period. In contrast, hTERT-HME1 normal breast epithelial cells maintained baseline proliferation patterns when exposed to identical Ac-Gb4 concentrations, with proliferation curves remaining parallel between treated and control groups throughout the observation period (Figure 3C,D). These findings indicate selective anti-proliferative effects of Ac-Gb4 against malignant cells while preserving normal cell function, suggesting potential therapeutic applications with minimal impact on healthy tissue.

### 2.5. Induction of Apoptosis by Ac-Gb4 in Breast Cancer Cells

**Figure 4:**
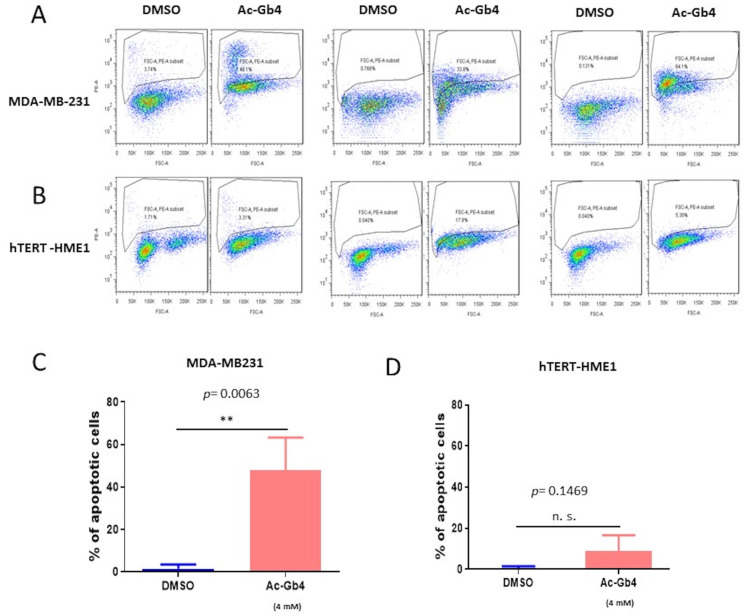
Evaluating Apoptotic Effects of Ac-Gb4 in Breast Cancer and Normal Cells. Flow cytometric analysis of Ac-Gb4-induced apoptosis in breast cancer and normal epithelial cells. (**A**) Representative flow cytometry dot plots showing apoptotic cell populations in MDA-MB-231 breast cancer cells treated with DMSO (control) or Ac-Gb4 (4 mM). The Annexin V-positive cell population in the selected area of the plot indicate apoptotic cells. Three independent experiments were performed. (**B**) Representative flow cytometry dot plots of hTERT-HME1 normal breast epithelial cells treated with DMSO or Ac-Gb4 (4 mM) in three independent experiments. (**C**) Quantitative analysis of apoptotic cells in MDA-MB-231 cells treated with DMSO or Ac-Gb4 (4 mM), demonstrating a significant increase in apoptosis (*p* = 0.0063, ** *p* < 0.01). (**D**) Quantitative analysis of apoptotic cells in hTERT-HME1 cells treated with DMSO or Ac-Gb4 (4 mM), showing no significant difference (*p* = 0.1469, n.s.). Data are presented as mean ± SEM from three independent experiments.

Further investigation into the cellular response to Ac-Gb4 treatment revealed significant differences in apoptotic outcomes between cancer and normal cell populations. Flow cytometric analysis demonstrated that MDA-MB-231 cells treated with 4 mM Ac-Gb4 exhibited a substantial increase in the apoptotic percentage compared to that of the DMSO-treated controls (Figure 4A,C). The apoptotic response in these cancer cells occurred in a time-dependent manner, indicating a progressive impact on cell viability following Ac-Gb4 exposure. Analysis of cellular morphology and membrane integrity markers further confirmed the induction of programmed cell death in the cancer cell population.

Examination of hTERT-HME1 normal breast epithelial cells under identical treatment conditions revealed markedly different responses. These normal cells showed no significant changes in the apoptotic rate when exposed to 4 mM Ac-Gb4, maintaining cellular integrity and viability comparable to the control conditions (Figure 4B,D). The differential response between cancer and normal cells suggests specific molecular mechanisms underlying the selective cytotoxicity of Ac-Gb4 toward malignant cells. This selective induction of cell death in cancer cells while preserving normal cell viability represents a crucial characteristic for potential therapeutic applications.

### 2.6. Impact of Ac-Gb4 on Cancer Cell Invasion Capability

**Figure 5:**
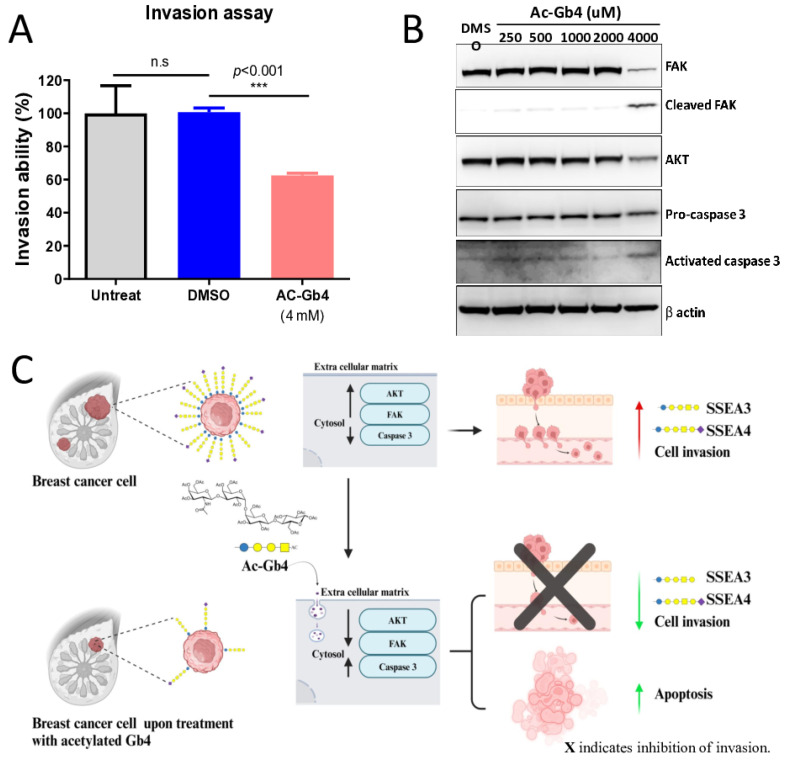
Exploring the Mechanism of Ac-Gb4 on Cancer Cell Invasion and Associated Signaling Pathway. Investigation of Ac-Gb4-mediated invasion suppression and its molecular mechanism in breast cancer cells. (**A**) Matrigel-Transwell invasion assay demonstrating significant reduction in MDA-MB-231 cell invasiveness following Ac-Gb4 (4 mM) treatment compared to that of untreated and DMSO controls. Results are presented as percentage of invasion ability normalized to untreated control and represented as *p*-value. *** *p* < 0.001 show the comparison of Ac-Gb4 with DMSO controls. (**B**) Western blot analysis showing dose-dependent effects of Ac-Gb4 (250–4000 μM) on key signaling proteins in MDA-MB-231 cells. Analysis included FAK, cleaved FAK, AKT, pro-caspase 3, and activated caspase 3, with β-actin as loading control. (**C**) Proposed mechanistic model illustrating the role of Ac-Gb4 in breast cancer cell invasion suppression. The upper panel illustrates the regulatory mechanisms of glycosphingolipid-mediated signaling in breast cancer cells, where surface expression of stage-specific embryonic antigens (SSEA3 and SSEA4) maintains active FAK/AKT signaling pathways and suppressed caspase 3 activation, promoting cellular invasion. The lower panel demonstrates the molecular mechanism of Ac-Gb4-mediated invasion suppression, wherein Ac-Gb4 treatment induces a significant reduction in SSEA3/SSEA4 surface expression, leading to downregulation of FAK and AKT signaling cascades. This signaling modulation triggers the activation of caspase 3-dependent apoptotic pathway, resulting in diminished cellular invasion capacity and enhanced programmed cell death. The diagram emphasizes the interconnected nature of glycosphingolipid expression, cellular signaling, and the phenotypic outcomes of invasion and survival in breast cancer cells.

Analysis of the invasion capabilities revealed that Ac-Gb4 treatment substantially impacted the invasive behavior of MDA-MB-231 breast cancer cells. Treatment with increasing concentrations of Ac-Gb4 (0.25 mM to 4 mM) demonstrated a dose-dependent reduction in cellular invasion ability. At the highest concentration tested (4 mM), Ac-Gb4 treatment led to a significant inhibition of invasion compared to the untreated and DMSO controls (Figure 5A). Quantitative analysis confirmed a progressive decrease in invasion capability that correlated directly with increasing Ac-Gb4 concentration. This relationship between Ac-Gb4 concentration and invasion inhibition suggests direct modulation of cellular mechanisms crucial for cancer cell invasion. These findings hold particular significance given the aggressive nature of MDA-MB-231 cells and their typically high invasive potential, indicating potential therapeutic relevance for preventing cancer cell invasion and metastasis.

### 2.7. Molecular Mechanism Analysis of Ac-Gb4-Induced Effects

To evaluate the molecular mechanisms underlying the observed cellular responses to Ac-Gb4 treatment, we examined key signaling proteins involved in cell survival and apoptotic pathways. Western blot analysis was performed on MDA-MB-231 cells treated with increasing concentrations of Ac-Gb4 (0.25 to 4 mM) for 24 h. The expression levels of focal adhesion kinase (FAK), AKT, and caspase-3, critical regulators of cell survival and apoptosis, were assessed. Analysis revealed dose-dependent changes in these signaling molecules (Figure 5B). At the highest concentration of Ac-Gb4 (4 mM), a marked decrease in total FAK protein levels was observed, accompanied by the appearance of cleaved FAK fragments. Similarly, AKT expression levels decreased substantially at 4000 µM (4 mM) Ac-Gb4, indicating disruption of the survival signaling pathways. The apoptotic response was further confirmed by the conversion of pro-caspase 3 to its activated form, with increased levels of activated caspase-3 observed at 4000 µM (4 mM) Ac-Gb4 treatment.

These molecular changes align with the previously observed cellular responses, particularly the induction of apoptosis and reduction in cell invasion capability. The observed FAK cleavage establishes a molecular link to reduced invasion capacity in Ac-Gb4-treated cells, as FAK functions as a critical mediator in cellular adhesion and invasion processes. Additionally, the decreased AKT expression and increased caspase-3 activation demonstrate the engagement of apoptotic pathways, supporting the observed cell death in cancer cells. These findings suggest that Ac-Gb4 exerts anti-cancer effects through the simultaneous modulation of signaling pathways that target both survival signals and apoptotic mechanisms. The dose-dependent nature of these molecular changes indicates a directed and specific effect of Ac-Gb4 on these cellular pathways, providing mechanistic insight into its selective cytotoxicity toward cancer cells.

The present study demonstrates that Ac-Gb4 effectively modulates multiple signaling pathways simultaneously to achieve anti-cancer effects by targeting both survival signals and apoptotic mechanisms in breast cancer cells (Figure 5C). Flow cytometric analysis revealed selective induction of apoptosis in MDA-MB-231 breast cancer cells while maintaining normal breast epithelial cell viability. Western blot analysis demonstrated that Ac-Gb4 treatment induces FAK cleavage, reduces AKT expression, and activates caspase-3, establishing the molecular mechanism for decreased cancer cell invasion and survival. These molecular changes align with the functional analyses demonstrating decreased cell invasion capability in a dose-dependent manner. In our previous study, we observed that FAK and AKT proteins are cleaved by caspase 3 when cells are treated with knockdown of the β3GalT5 enzyme [19]. Therefore, in the current study, we focused on the total protein expression levels of FAK and AKT, as their cleavage is induced by caspase-3 activation. We observed that Ac-Gb4 suppresses TNBC progression by modulating the FAK/AKT pathway. Taken together, these results indicate that Ac-Gb4 represents a promising therapeutic candidate for breast cancer treatment by targeting cancer cells through the modulation of critical signaling pathways involved in cell survival and invasion.

## 3. Discussion

The findings from this study underscore the potential of Ac-Gb4 in selectively targeting breast cancer cells, effectively reducing viability, promoting apoptosis, and suppressing invasive behavior. The selective glycolipid targeting observed may arise from inherent differences in glycolipid composition between malignant and normal cells, with cancer cells exhibiting greater susceptibility to glycolipid-targeted interventions. The differing responses of MDA-MB-231 cancer cells and normal hTERT-HME1 cells to Ac-Gb4 treatment demonstrate how cancer-specific membrane characteristics enhance lipophilic Ac-Gb4 binding and uptake to achieve selective cytotoxicity. The observed modulation of FAK, AKT, and caspase-3 provides mechanistic insights into the anti-cancer effects of Ac-Gb4. In our previous study, FAK downregulation and subsequent cleavage in MDA-MB-231 cells align with reduced adhesion and invasion capabilities, while decreased AKT expression corresponds to impaired survival signaling [19]. The activation of caspase-3 further consolidates the apoptotic pathway as a critical outcome of Ac-Gb4 treatment in breast cancer cells. The present study investigated the effects of acetylated Globotetraose (Ac-Gb4) on breast cancer cells, specifically focusing on the MDA-MB-231 cell line and hTERT-HME1 immortalized normal breast epithelial cells. The findings reveal several important aspects regarding the potential therapeutic applications of Ac-Gb4 in breast cancer treatment and its differential effects on cancerous versus normal cells. One of the most significant findings of this study is the distinct response patterns observed between cancerous MDA-MB-231 cells and normal hTERT-HME1 immortalized breast epithelial cells when treated with Ac-Gb4. The proliferation assays demonstrated that Ac-Gb4 treatment (4 mM) had a more pronounced inhibitory effect on MDA-MB-231 cells compared to that on hTERT-HME1 cells. The establishment of 4 mM as an effective concentration for subsequent experiments appears well-justified based on the observed responses, though future studies might benefit from exploring the molecular mechanisms underlying this dose-dependent response. The utilization of 4 mM Ac-Gb4 in the current study presents significant challenges for therapeutic applications, primarily due to the considerably higher concentration compared to typical drug concentrations used in clinical settings. Most successful therapeutic agents operate in the micromolar or even nanomolar range, making the 4 mM concentration a potential barrier to clinical translation.

The requirement for high drug concentrations limits clinical applications because of potential toxicity issues and challenges in achieving therapeutic effectiveness within the body [29]. To address these challenges, several promising strategies could be implemented [30]. Chemical modification of the Ac-Gb4 molecule represents a primary approach, focusing on enhanced lipophilicity through strategic modifications to improve membrane permeability and cellular uptake efficiency. This can be achieved by optimizing the acetylation pattern and introducing targeted lipophilic moieties [31]. Additionally, incorporating cancer-specific targeting groups, such as antibodies or peptides, could improve the molecular specificity for cancer cells, potentially reducing the required concentration for therapeutic effects [32].

Novel formulation approaches offer another avenue for improvement through the development of specialized delivery systems [33]. Advanced nanocarrier systems, including liposomal formulations and polymeric nanoparticles, could enhance drug solubility and cellular uptake while providing controlled release mechanisms [34]. These smart delivery platforms could enable targeted release at tumor sites, improving local drug concentrations while maintaining lower systemic levels [35,36]. This approach could significantly enhance bioavailability and reduce the overall concentration needed for therapeutic efficacy. The integration of these chemical modifications with advanced delivery systems presents the most promising path forward for improving the therapeutic profile of Ac-Gb4. Such improvements would address the current limitations posed by the high concentration requirement while maintaining or enhancing the anti-cancer effects. Future research should focus on optimizing these approaches to develop a more clinically viable version of Ac-Gb4-based therapy.

The apoptosis assay results provide compelling evidence for the anti-cancer mechanism of Ac-Gb4. The statistically significant increase in apoptotic cells observed in MDA-MB-231 cells compared to the non-significant changes in hTERT-HME1 cells further supports the selective nature of effects induced by Ac-Gb4. This differential apoptotic response between cancerous and normal cells represents a crucial finding that warrants further investigation into the molecular pathways involved. Several potential mechanisms might explain this selective induction of apoptosis. The varying responses could be attributed to differences in glycolipid expression patterns between cancer and normal cells, as cancer cells often exhibit altered glycolipid profiles [10], which might make them more susceptible to Ac-Gb4-mediated effects. Furthermore, the stronger apoptotic response in MDA-MB-231 cells suggests that Ac-Gb4 might be activating specific death receptor pathways or mitochondrial-mediated apoptosis pathways that are more readily triggered in cancer cells [26]. The acetylated nature of Gb4 might also lead to different interactions with membrane components in cancer cells versus normal cells, potentially disrupting cancer cell-specific membrane organization or signaling platforms.

The selective cytotoxicity observed with Ac-Gb4 treatment demonstrates a promising therapeutic window based on fundamental differences between cancer and normal cells in their glycolipid biology. Dose-response studies from 0.25 mM to 4 mM showed concentration-dependent effects, with 4 mM established as an effective concentration for experimental studies. The apoptosis assay revealed significant selective effects, with MDA-MB-231 cells showing a marked apoptotic response, while hTERT-HME1 cells remained largely unaffected. This differential response stems from a key biological distinction: MDA-MB-231 cancer cells express glycolipids on their surface and critically depend on these glycolipids to maintain their growth and survival, whereas normal cells neither express nor require glycolipids for cellular maintenance. When treated with Ac-Gb4, cancer cells show a significant response because the treatment interferes with their essential glycolipid-dependent processes, while normal cells remain unaffected due to their glycolipid-independent nature. This inherent difference in glycolipid biology between cancer and normal cells not only explains the observed selective cytotoxicity in the experimental results but also suggests potential therapeutic advantages for clinical applications.

A notable discovery shows that Ac-Gb4 effectively inhibits the invasion capability of MDA-MB-231 cells. The invasion assay results demonstrate a clear dose-dependent relationship, with higher concentrations of Ac-Gb4 leading to a greater inhibition of the invasive capacity. This finding has particular relevance given that MDA-MB-231 is a highly aggressive, triple-negative breast cancer cell line known for its invasive properties. In our previous study, we conducted an invasion assay to assess the role of globo-series glycosphingolipids and β3GalT5 in breast cancer [19]. This work demonstrated that modulation of specific glycolipids could significantly impact invasion capabilities in breast cancer cells, a methodologically relevant finding that aligns with our current findings on Ac-Gb4 inhibitory effects on MDA-MB-231 cells. This effect suggests that Ac-Gb4 could be attributed to several potential mechanisms. Ac-Gb4 might affect the organization or dynamics of the cytoskeleton, which is crucial for cell migration and invasion. The treatment might also influence the expression or activity of matrix metalloproteinases, key enzymes involved in cancer cell invasion. Additionally, modifications in cell surface glycolipids could alter the capability of cells to interact with extracellular matrix components.

Although the results demonstrate clear invasion inhibition by Ac-Gb4, the exact molecular mechanism requires further investigation. Multiple pathways might contribute to this anti-cancer effect including cytoskeletal changes, matrix metalloproteinase activity modifications, and altered cell–matrix interactions. Notably, previous studies have shown that globo-series Gb4 promotes cell proliferation through ERK pathway activation, suggesting that Ac-Gb4 might achieve its anti-cancer effects by disrupting this ERK-mediated proliferation signal [37]. The complexity of these potential mechanisms including ERK pathway interference creates significant challenges for optimizing treatment strategies and anticipating resistance development without identifying the primary molecular pathway.

While the current study provides valuable insights, several limitations and areas for future investigation should be acknowledged. Additional studies are needed to fully understand the molecular mechanisms underlying the selective effects of Ac-Gb4 on cancer cells, including investigation of specific apoptotic pathways, analysis of changes in membrane organization, and examination of alterations in cellular signaling pathways. The current study focused on relatively short-term effects, and long-term studies would be valuable to assess the development of resistance, sustained anti-cancer effects, and impact on normal cell function over extended periods. Future studies should also explore the effects on other breast cancer subtypes, potential applications in other cancer types, and combination treatments with established therapeutic agents. The promising in vitro results warrant investigation in animal models to assess systemic distribution and metabolism, anti-tumor effects in vivo, and safety and tolerability.

The findings from this study have several potential clinical implications. The selective effects of Ac-Gb4 on cancer cells suggest its potential as a novel therapeutic agent, particularly for aggressive breast cancers. The differential response between cancer and normal cells indicates potential for its development as a targeted therapy with reduced side effects. Moreover, the distinct mechanism of action might make Ac-Gb4 a valuable addition to existing treatment regimens, potentially enhancing their effectiveness.

## 4. Materials and Methods

### 4.1. Chemicals and Reagents

Globotetraose (Gb4) was acquired from standard biochemical suppliers; its acetylated form (Ac-Gb4) was generously provided as a gift by Dr. Shie, Jiun-Jie at Institute of Chemistry, Academia Sinica, Taiwan; and their purity was confirmed by high-performance liquid chromatography (HPLC). For cellular treatments, Ac-Gb4 was dissolved in dimethyl sulfoxide (DMSO) as a stock solution at 10 mM and stored at −20 °C. All antibodies, including those targeting glycolipid markers SSEA3 (MC631), SSEA4 (MC81370), and Globo-H (VK 9 clone), were purchased from Biolegend (San Diego, CA, USA). β-actin (Catalog No. MABT523) was purchased from Sigma-Aldrich (St. Louis, MO, USA). Dulbecco’s Modified Eagle Medium (Catalog No. 11965-175) purchased from Invitrogen (Carlsbad, CA, USA). Calicut AM (Catalog No. C3099) was purchased from Thermo Fisher Scientific Inc., Waltham, MA, USA. CCK8 (product number: CK-04) was purchased from Dojindo (Kumamoto, Japan). The hTERT-HME1 (ME16C) cell line (Catalog No. CRL-4010) and MDA-MB-231 cells (Catalog No. CRM-HTB-26) were purchased from Sigma-Aldrich (St. Louis, MO, USA). These signaling proteins such as FAK (Catalog No. sc-271126) were purchased from Santa Cruz Biotechnology (Dallas, TX, USA), and AKT (Catalog No. 9272), and caspase-3 (Catalog No. 9662) was purchased from Cell Signaling Technology (Danvers, MA, USA) and validated by the manufacturer for use in Western blot and flow cytometric applications. Additional cell culture reagents, such as fetal bovine serum (Catalog No. 26140-079, Gibco (Waltham, MA USA), a brand of Thermo Fisher Scientific Inc., Waltham, MA, USA), were sourced from certified vendors and were used as recommended by the supplier.

### 4.2. Expression and Survival Analysis

The gene expression of B3GALT5 in breast cancer was studied using GEPIA2 (http://gepia2.cancer-pku.cn/#survival, accessed on 13 November 2024) platform. A detailed procedure to run the expression analysis is explained in Figure S1. Relative expression was analyzed through box plot in normal and breast cancer samples. The sample information is collective data from TCGA and GTEx platforms and the accession along with data set details (Figures S2 and S3, Tables S1 and S2). The survival analysis of patients was performed by using Kaplan–Meier plotter platform. Relative survival analysis of distant metastases-free survival (DMFS) in patients with triple-negative breast cancer (ER-negative, PR-negative, and HER2-negative). The parameters for this survival analysis can be found in the link and in Figure S4.

### 4.3. Gene Enrichment Analysis

Using Correlation AnalyzeR (https://gccri.bishop-lab.uthscsa.edu/shiny/correlation-analyzer/, accessed on 13 November 2024) as explained in Figure S5, the B3GALT5 gene correlation analysis was carried out to understand the involvement of closely related genes in breast cancer patients, and the scatter plots of specific genes were obtained through GEPIA2, as displayed in Figure S6.

### 4.4. Drug-Likeness and ADMET Analysis

The drug-likeness properties and ADMET profiling of Ac-Gb4 were carried out using admetSAR 3.0 (https://lmmd.ecust.edu.cn/admetsar3/predict.php, accessed on 3 April 2024). The analysis was conducted under default parameters.

### 4.5. Molecular Docking Analysis

The molecular docking analysis tool MOE15 (https://www.chemcomp.com, accessed on 10 August 2024) was used to predict the best interacting of Ac-Gb4 with Beta-1,3-galactosyltransferase 5. B3GALT5 structure was retrieved from AlphaFold (https://alphafold.ebi.ac.uk/entry/Q9Y2C3, accessed on 24 June 2024) in PDB format. B3GALT5 were optimized by removing water from the protein surface which hinders the binding of ligands by using MOE 15. Energy minimization step and three-dimensional protonation were performed. To predict active sites of all receptor proteins, MOE protocol was followed. Using the site finder option in MOE, the active sites were identified for analysis. Docking analysis was carried out by setting parameters (rescoring 1, London dG 10). The best interacting ligand and receptor were selected on basis of S-score and binding interaction [38].

### 4.6. Cell Lines and Culture Conditions

The human breast cancer cell line MDA-MB-231 and the immortalized non-cancerous human breast epithelial cell line hTERT-HME1 were cultured in Dulbecco’s Modified Eagle Medium (DMEM, Gibco) supplemented with 10% FBS, 100 U/mL penicillin, and 100 μg/mL streptomycin (Gibco). Cells were maintained at 37 °C in a humidified atmosphere of 5% CO_2_. Cultures were routinely monitored for mycoplasma contamination and passaged at 80% confluence. For all experiments, cells were seeded at a density of 1 × 10^5^ cells per well in 6-well plates and allowed to adhere overnight before treatment.

### 4.7. Acetylated Globotetraose (Ac-Gb4) Treatment

Cells were treated with Ac-Gb4 at concentrations ranging from 0.25 to 4 mM, with DMSO as a vehicle control. Treatment was carried out for 24 h for immediate cell viability assays, while longer durations (up to 96 h) were used to assess effects on cell proliferation, invasion, and apoptosis. Control groups received equivalent concentrations of DMSO without Ac-Gb4.

### 4.8. Flow Cytometry Analysis of Glycolipid Expression

To determine glycolipid expression levels, flow cytometry was performed using antibodies against SSEA3, SSEA4, and Globo-H. Cells were harvested, washed in phosphate-buffered saline (PBS), and incubated with primary antibodies for 30 min at room temperature. Following primary incubation with Alexa Fluor-conjugated antibodies for another 30 min, cells were washed and subjected to flow cytometry analysis. Data acquisition was performed using a BD FACSCanto II cytometer, and fluorescence intensity was analyzed with FlowJo software (version 10.10). Quantitative analysis involved the median fluorescence intensity (MFI) for each marker, comparing Ac-Gb4-treated cells to controls.

### 4.9. Cell Viability and Proliferation Assays

Cell viability and proliferation were evaluated using both CCK8 assay (Dojindo) and direct cell counting methods. The CCK8 assay measured cell viability through absorbance readings at 450 nm minus 690 nm background. Proliferation was monitored every 24 h for 96 h after treatment where cell numbers were normalized to initial seeding density. The combination of absorbance data and cell counts provided comprehensive analysis of Ac-Gb4 effects on cell growth.

### 4.10. Apoptosis Assay

Apoptotic induction was evaluated using Annexin V/propidium iodide (PI) staining (eBioscience, Thermo Fisher Scientific, Waltham, MA, USA). After Ac-Gb4 treatment, cells were incubated with Annexin V-FITC and PI according to the manufacturer’s instructions. Flow cytometric analysis of apoptotic cells (Annexin V+/PI-) was conducted, and results were statistically analyzed to determine significance between treated and control groups.

### 4.11. Invasion Assay

Invasion capability was measured using Matrigel (Corning Matrigel)-coated Transwell chambers (Falcon, New York, NY, USA). MDA-MB-231 cells treated with varying concentrations of Ac-Gb4 were seeded in the upper chamber, while the lower chamber contained 10% FBS as a chemoattractant. After 24 h incubation, non-invading cells on the upper membrane surface were removed with cotton swabs. The invaded cells on the lower surface were fixed and stained with Calicin AM (Invitrogen). Quantification was performed by measuring fluorescence intensity at excitation/emission wavelengths of 485/530 nm using an ELISA reader. The invasion inhibition rate was calculated by comparing fluorescence readings of treated samples to those of DMSO controls and expressed as percentage of control.

### 4.12. Western Blot Analysis

To assess signaling pathway alterations, total protein was extracted from treated cells using RIPA buffer (50 mM Tris-HCl, pH 7.4, 150 mM NaCl, 1% NP-40, 0.5% sodium deoxycholate, 0.1% SDS) supplemented with protease and phosphatase inhibitors. Cells were lysed on ice for 30 min, followed by centrifugation at 14,000× *g* for 15 min at 4 °C to remove cell debris. The supernatant was collected, and concentrations were quantified by the BCA assay [39]. Equal amounts of protein were subjected to 10% SDS-PAGE and transferred to polyvinylidene difluoride (PVDF) membranes using a wet transfer system at 100 V for 1 h at 4 °C. Membranes were then blocked with 5% non-fat dry milk in Tris-buffered saline with 0.1% Tween-20 (TBS-T) for 1 h at room temperature to prevent non-specific binding. Following blocking, membranes were blocked and incubated overnight with primary antibodies against FAK, AKT, caspase-3, and activated caspase-3, followed by horseradish peroxidase-conjugated secondary antibodies for 1 h at room temperature. Immunoreactive bands were visualized using enhanced chemiluminescence (ECL) reagents, band intensities were quantified with ImageJ software (version number 1.53t), and protein expression was normalized to loading controls (β-actin) [40].

### 4.13. Statistical Analysis

Results are expressed as the mean ± SEM. Analysis of variance (ANOVA) and t-tests were performed for comparative analysis of the different groups. The level of significance was considered in terms of the *p*-value: * *p* < 0.05, ** *p* < 0.01, *** *p* < 0.001, and **** *p* < 0.0001 was considered statistically significant. Graph generation and data analysis were performed using GraphPad Prism (7.01).

## 5. Conclusions

In conclusion, this study provides compelling evidence for Ac-Gb4 as a promising therapeutic agent that selectively targets breast cancer cells through multiple mechanisms. These include the modulation of glycolipid expression, induction of apoptosis, and suppression of invasive potential. Ac-Gb4 demonstrates considerable potential for developing targeted cancer therapies by selectively interacting with glycolipids specific to malignant cells while showing limited effects on normal breast epithelial cells. The successful synthesis and cellular uptake of Ac-Gb4 combined with its differential effects between cancer and normal cells significantly advance our understanding of glycolipid-based therapeutic approaches. These findings contribute to our knowledge of glycolipid biology in cancer and establish a foundation for developing novel therapeutic strategies. Future research should focus on elucidating precise mechanisms of action, optimizing delivery methods, and evaluating efficacy in in vivo models to further explore the clinical applicability of Ac-Gb4 in breast cancer treatment.

## Data Availability

The data presented in this study will be available on request from the corresponding author.

## Supplementary Materials

The following supporting information can be downloaded at: https://www.mdpi.com/article/10.3390/ijms252413353/s1.
