# Supplementary material for: Acetylated Globotetraose (Ac-Gb4) Suppresses Triple-Negative Breast Cancer Through FAK/AKT Signaling Pathway
Source: Int J Mol Sci. 2024 Dec 12;25(24):13353. doi: 10.3390/ijms252413353 (PMC11677054; doi:10.3390/ijms252413353)
Supplement: Supplementary file 1 [file ijms-25-13353-s001.zip › ijms-3322479-supplementary.pdf]

Supplementary Materials File

This file includes:

Figure S1. Methodology for Accessing B3GALT5 Gene Expression in Breast Cancer (BRCA) Samples Using GEPIA 2.
Figure S2. The detailed dataset information of BRCA samples from the cancer proteome atlas which is from the data base available on TCGA portal.
Figure S3. Expression of B3GALT5 enzyme in samples from GTEx database with (Accession phs000424.v10.p2).
Figure S4. Kaplan-Meier Plotter details for triple negative breast cancer survival analysis.
Figure S5. Correlation analysis workflow for B3GALT5 in mammary tissue.
Figure S6. Top 10 genes that show correlation with B3GALT5 in breast cancer.
Figure S7. Drug-likeness properties of Ac-Gb4.
Figure S8. Chemical structure of Ac-Gb4 used for molecular docking.
Figure S9. Flow cytometry analysis of globo-series glycosphingolipid expression in breast cancer cell line.
Table S1. The detailed dataset information of BRCA samples from TCGA portal.
Table S2. Data Source: GTEx Analysis Release V10 (dbGaP Accession phs000424.v10.p2).
Table S3. log(inhibitor) vs. normalized response.
Table S4. Correlation of top 10 genes with B3GALT5 gene in breast cancer tissue.
Table S5. Top 10 genes with least correlation with B3GALT5 gene in breast cancer tissue.
Table S6. Absorption, metabolism and toxicity-related drug-like properties of Ac-Gb4.
Table S7. Docking analysis of Beta-1,3-galactosyltransferase with Ac-Gb4.

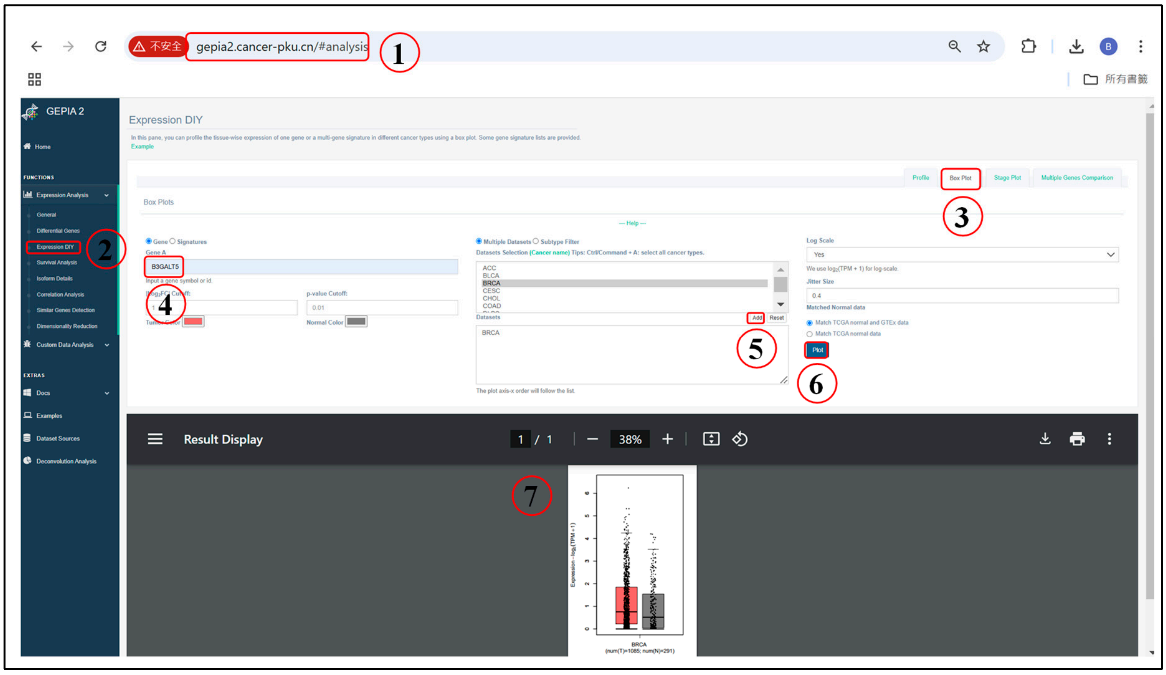

Figure S1. Methodology for Accessing B3GALT5 Gene Expression in Breast Cancer (BRCA) Samples Using GEPIA 2. To analyze B3GALT5 gene expression in BRCA samples, follow these steps: (1) Open the URL at GEPIA 2 (http://gepia2.cancer-pku.cn/#analysis, accessed on 2 November 2024), (2) From the left menu, select Expression DIY under Expression Analysis, (3) Choose Box Plot as the plot type, (4) Enter B3GALT5 as the target gene, (5) Select BRCA as the cancer type and click Add, (6) Click Plot to generate the visualization, and (7) Download the resulting plot as a PDF if needed.

Supplementary Materials File

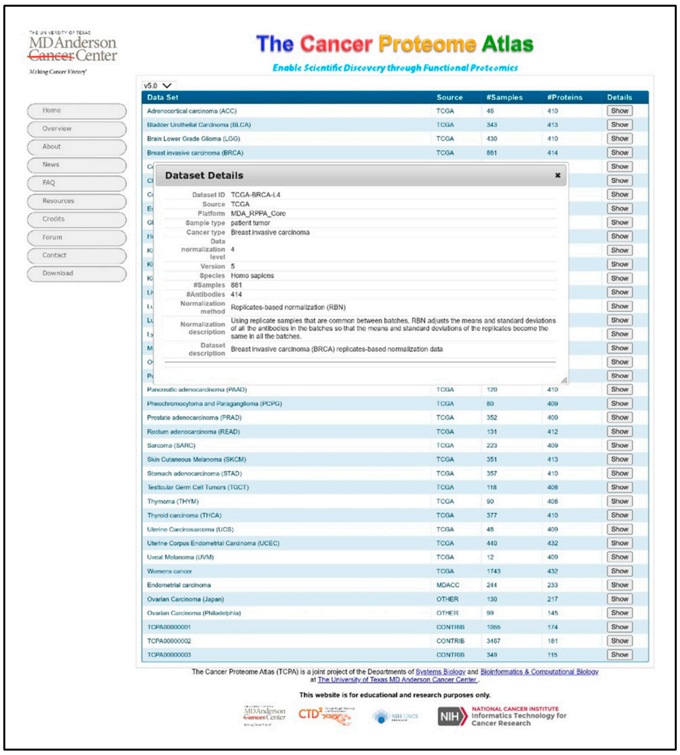

Figure S2: The detailed dataset information of BRCA samples from the cancer proteome atlas which is from the data base available on TCGA portal.

Table S1. The detailed dataset information of BRCA samples from TCGA portal.

Dataset ID	TCGA-BRCA-L4
Source	TCGA
Platform	MDA_RPPA_Core
Sample type	patient tumor
Cancer type	Breast invasive carcinoma
Data normalization level	4
Version	5
Species	Homo sapiens
Samples	881
Antibodies	414
Normalization method	Replicates-based normalization (RBN)
Normalization description	Using replicate samples that are common between batches, RBN adjusts the means and standard deviations of all the antibodies in the batches so that the means and standard deviations of the replicates become the same in all the batches.
Dataset description	Breast invasive carcinoma (BRCA) replicates-based normalization data

Supplementary Materials File

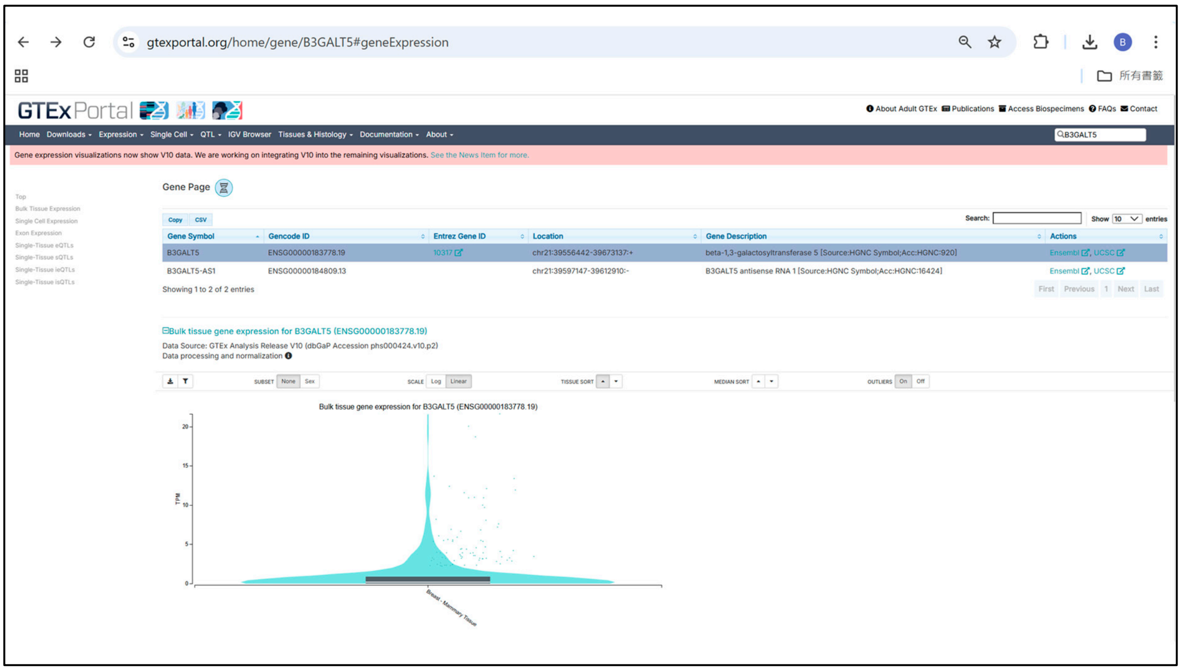

Figure S3. Expression of B3GALT5 enzyme in samples from GTEx database with (Accession phs000424.v10.p2).

Table S2. Data Source: GTEx Analysis Release V10 (dbGaP Accession phs000424.v10.p2).

Gene Symbol	Gencode ID	Entrez Gene ID	Location	Gene Description
B3GALT5	ENSG00000183778.19	10317	chr21:39556442-39673137:+	beta-1,3-galactosyltransferase 5 [Source:HGNC Symbol;Acc:HGNC:920]

Supplementary Materials File

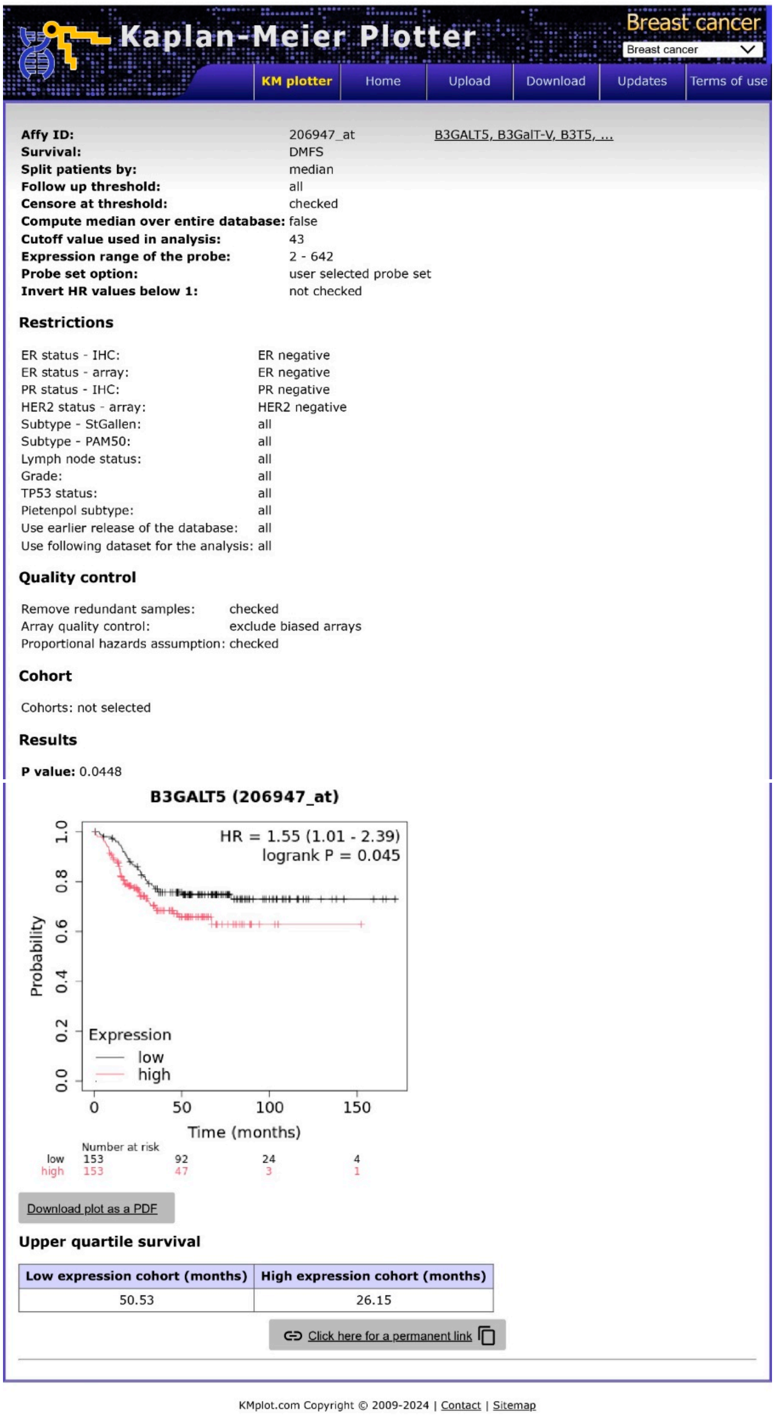

Kaplan-Meier Plotter
Breast cancer
Breast cancer
KM plotter | Home | Upload | Download | Updates | Terms of use

Affy ID: 206947_at B3GALT5, B3GalT-V, B3T5, ...
Survival: DMFS
Split patients by: median
Follow up threshold: all
Censore at threshold: checked
Compute median over entire database: false
Cutoff value used in analysis: 43
Expression range of the probe: 2 - 642
Probe set option: user selected probe set
Invert HR values below 1: not checked

Restrictions

ER status - IHC: ER negative
ER status - array: ER negative
PR status - IHC: PR negative
HER2 status - array: HER2 negative
Subtype - StGallen: all
Subtype - PAM50: all
Lymph node status: all
Grade: all
TP53 status: all
Pietenpol subtype: all
Use earlier release of the database: all
Use following dataset for the analysis: all

Quality control

Remove redundant samples: checked
Array quality control: exclude biased arrays
Proportional hazards assumption: checked

Cohort

Cohorts: not selected

Results

P value: 0.0448

Download plot as a PDF

Upper quartile survival

Low expression cohort (months)	High expression cohort (months)
50.53	26.15

Click here for a permanent link

KMplot.com Copyright © 2009-2024 | Contact | Sitemap

Figure S4. Kaplan-Meier Plotter details for triple negative breast cancer survival analysis.

Supplementary Materials File

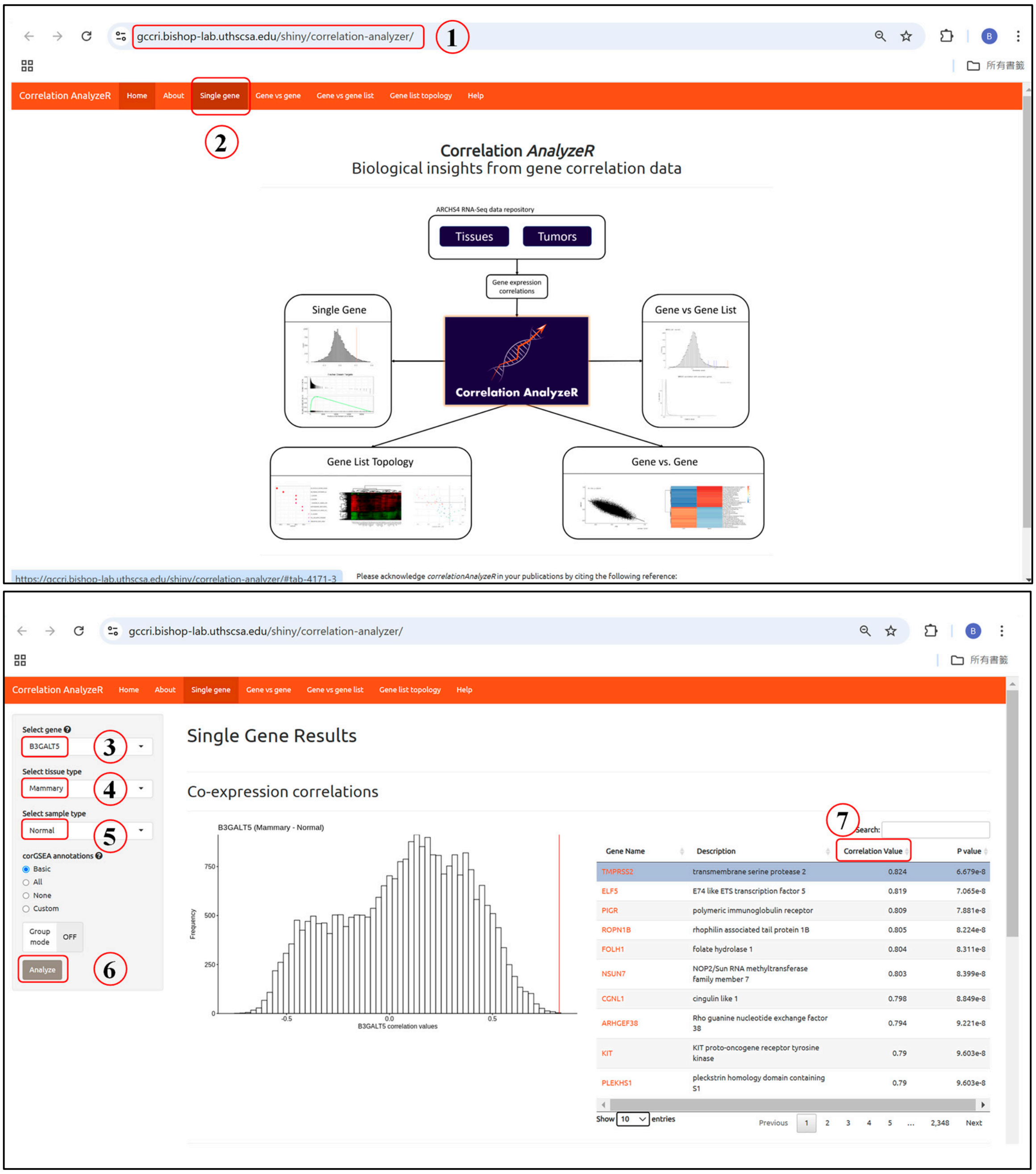

Figure S5. Correlation analysis workflow for B3GALT5 in mammary tissue. The figure illustrates the steps to analyze B3GALT5 gene correlations using the GCCRI correlation analyzer tool. (1) Access the URL at GCCRI Correlation Analyzer (https://gccri.bishop-lab.uthscsa.edu/shiny/correlation-analyzer/, accessed on 2 November 2024), (2) Select Single Gene from the header options, (3) Choose the target gene B3GALT5, (4) Set the tissue type to Mammary, (5) Specify the sample type as Normal/Cancer, (6) Click on Analyze to run the analysis, and (7) Sort the resulting correlation values in descending order from high to low.

Supplementary Materials File

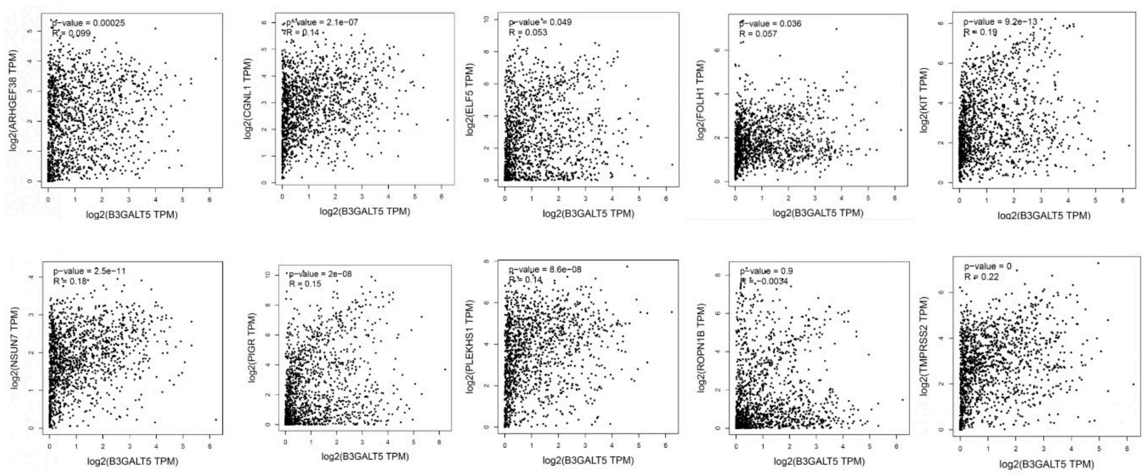

Figure S6. Top 10 genes that show correlation with B3GALT5 in breast cancer. The figure displays X-axis indicating the expression of B3GALT5 gene and Y-axis displaying the expression of the correlated gene. Correlation values were plotted using Pearson coefficient value.

Table S3. log(inhibitor) vs. normalized response.

**Parameter**	**Value**
**Best-Fit Values**	
$LogIC_{50}$	1.913
IC_{50}	81.85
**Standard Error**	
$LogIC_{50}$	0.1583
**95% Confidence Intervals**	
$LogIC_{50}$	1.474 to 2.352
IC_{50}	29.75 to 225.2
**Goodness of Fit**	
Degrees of Freedom	4
R^2	0.9348
Absolute Sum of Squares	383.6
Sy.x	9.793
**Number of Points Analyzed**	5

Supplementary Materials File

Table S4. Correlation of top 10 genes with B3GALT5 gene in breast cancer tissue.

Gene	Description	Correlation Value
TMPRSS2	Transmembrane serine protease 2	0.824
ELF5	E74 like ETS transcription factor 5	0.819
PIGR	Polymeric immunoglobulin receptor	0.809
ROPN1B	Rhophilin associated tail protein 1B	0.805
FOLH1	Folate hydrolase 1	0.804
NSUN7	NOP2/Sun RNA methyltransferase family member 7	0.803
CGNL1	Cingulin like 1	0.798
ARHGEF 38	Rho guanine nucleotide exchange factor 38	0.794
KIT	KIT proto-oncogene receptor tyrosine kinase	0.79
PLEKHS1	Pleckstrin homology domain containing S1	0.79

Supplementary Materials File

Table S5. Top 10 genes with least correlation with B3GALT5 gene in breast cancer tissue.

Gene	Description	Correlation Value
PROCR	Protein C receptor	-0.735
RGS20	Regulator of G protein signaling 20	-0.721
IL20RB	Interleukin 20 receptor subunit beta	-0.713
SHC1	SHC adaptor protein 1	-0.687
PTTG1	Pituitary tumor-transforming 1	-0.683
TRIM7	Tripartite motif containing 7	-0.683
RAET1E	Retinoic acid early transcript 1E	-0.676
S100A2	S100 calcium binding protein A2	-0.676
FSTL3	Follistatin like 3	-0.672
SRXN1	Sulfiredoxin 1	-0.668

**Molecular weight**	1253.3857
**Quantitative Estimate of Drug-likeness**	0.08
**logarithm of the partition coefficient**	-2.19
**Topological Polar Surface Area**	435.61
**Hydrogen bond donors**	1
**Hydrogen-bond acceptors**	34
**Number of rotatable bonds**	24
**Number of heteroatoms**	35
**Number of rings**	4
**Total number of atoms**	87

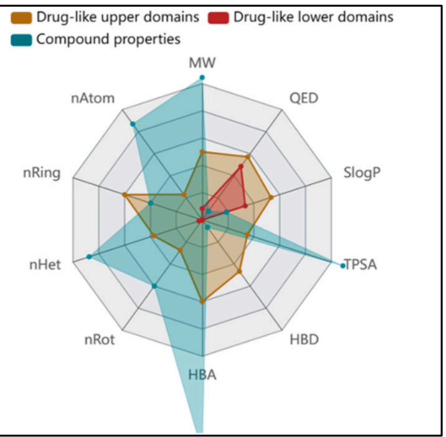

Figure S7. Drug-likeness properties of Ac-Gb4.

Supplementary Materials File

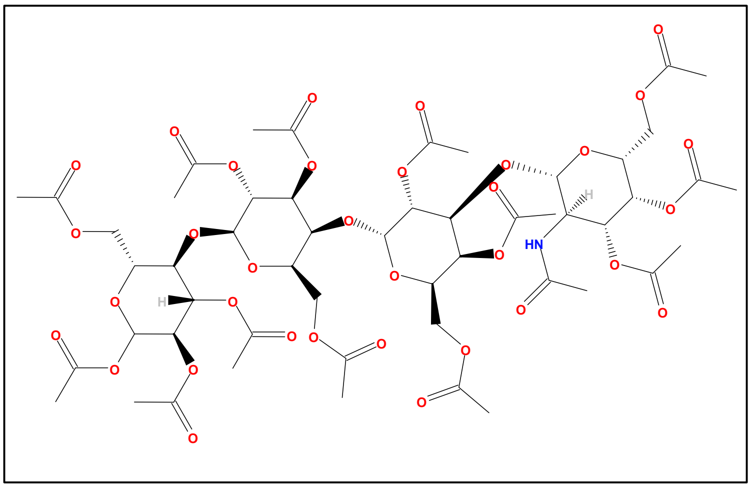

Figure S8. Chemical structure of Ac-Gb4 used for molecular docking.

Table S6. Absorption, metabolism and toxicity-related drug-like properties of Ac-Gb4.

**Absorption**					
BBB	HIA	CaCo2 permeability	PGS	PGI	ROCT
–	+	+	–	–	–
**Distribution**					
Subcellular localization			Mitochondria		
**Metabolism**					
CYP2D6 Substrate	CYP3A4 Substrate	CYP2C9 Substrate	CYP2C19 inhibition	CYP2D6 inhibition	CYP1A2 inhibition
–	+	–	–	–	–
CYP2C9 inhibition	CYP3A4 inhibition	CYP Inhibitory Promiscuity			
–	–	–			
**Toxicity**					
Ames toxicity			NAT		
Carcinogens			Non-carcinogenic		

BBB; blood–brain barrier, HIA; human intestinal absorption, PGS; P-glycoprotein substrate, PGI; P-glycoprotein inhibitor ROCT; renal organic cation transporter, NAT; non-Ames toxic. + and − represent presence and absence respectively.

Table S7. Docking analysis of Beta-1,3-galactosyltransferase with Ac-Gb4.

Receptor	COMPOUND	Interacting amino acids	S-score	No. of H-Bonds
B3GALT5	Ac-Gb4	Ans 187, Trp 207	-9.3	1

Supplementary Materials File

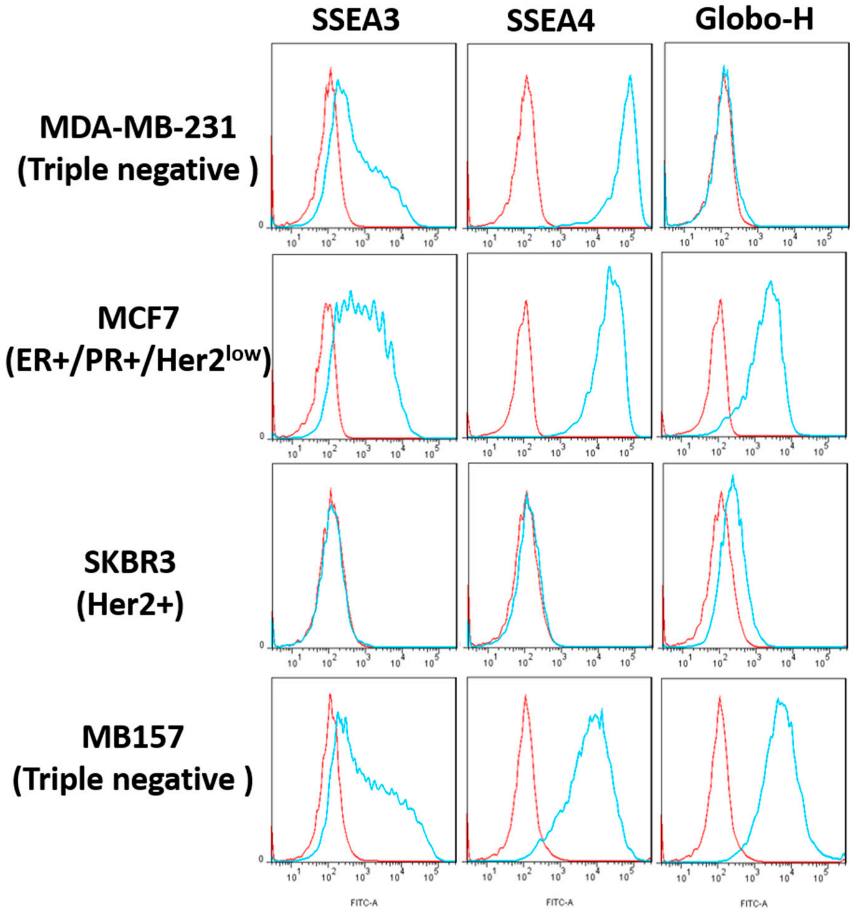

Figure S9. Flow cytometry analysis of globo-series glycosphingolipid expression in breast cancer cell line.
